# Embolization by Bullet Dislodged from the Heart

**DOI:** 10.21470/1678-9741-2017-0115

**Published:** 2017

**Authors:** Eduardo Cavalcanti Lapa Santos, Rodrigo Mezzalira Tchaick, Diogo Luiz de Magalhães Ferraz, João Paulo Segundo de Paiva Oliveira, Fernando Augusto Marinho dos Santos Figueira, George Augusto da Fonseca Carvalho Antunes Lima

**Affiliations:** 1 Division of Cardiovascular Surgery, Hospital Dom Helder Câmara (HDH), Cabo de Santo Agostinho, PE, Brazil.; 2 Instituto de Medicina Integral Professor Fernando Figueira (IMIP), Recife, PE, Brazil.; 3 Universidade Federal de Pernambuco (UFPE), Recife, PE, Brazil.

**Keywords:** Wounds, Penetrating, Heart, Wounds, Gunshot, Foreign-Body Migration, Carotid Arteries

## Abstract

Embolization by a dislodged projectile is a rare complication that may occur in cases of gunshot cardiac injuries. We report a case of a firearm projectile cardiac injury that evolved, with dislocation of the projectile during cardiac surgery, into embolization of the right external carotid artery.

**Table:** 

Abbreviations, acronyms & symbols	
CT	= Computed tomography
TTE	= Transthoracic echocardiogram

## INTRODUCTION

The main causes of penetrating injuries to the heart are gunshots and stabbings^[1]^. The mortality rate in such cases reaches 84%, in some series, being exsanguination the primary cause of death^[1]^. Young male adults are the main victims^[2]^. Embolization by a dislodged projectile is a rare complication that may occur in 0.3% of cases of gunshot cardiac injuries^[3]^.

## PATIENT CHARACTERIZATION AND DESCRIPTION OF THE TECHNIQUE EMPLOYED

A 26-year-old male patient was admitted to the emergency room due to injuries to the right hemithorax caused by a 12-gauge shotgun.

At first examination, the patient was hemodynamically stable, albeit presenting moderate dyspnea. No sounds were detectable by auscultation of the right hemithorax. Non-contrast computed tomography (CT) revealed the presence of metallic fragments in the thorax and abdomen, associated with right hemopneumothorax. Tube thoracostomy and laparotomy were performed. No abdominal visceral damage was found.

**Fig. 1:**
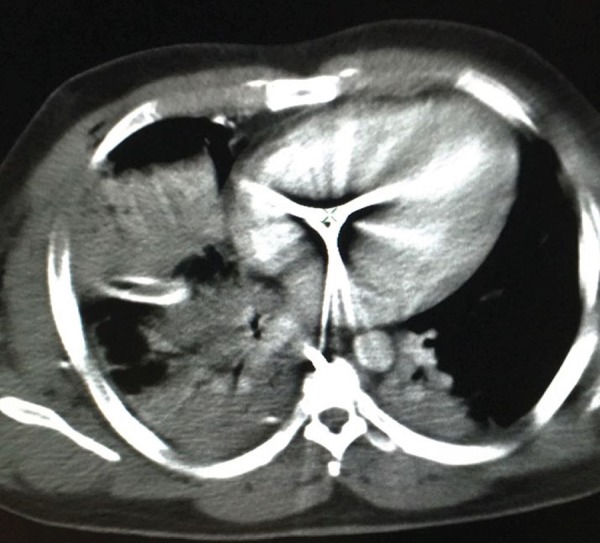
Cross-sectional chest tomography.

After clinical stabilization, the patient underwent further evaluation to precisely locate the projectiles in the mediastinum. Contrast-enhanced CT showed an image suggestive of a projectile fragment in intracardiac position (Figure 1). Transthoracic echocardiogram (TTE) was performed and showed a fixed, hyperechogenic image, measuring about 9x7 mm, located in the membranous septum region, and close to the septal cusp of the tricuspid valve. No intracardiac shunts were detected.

After careful consideration, the heart team decided to surgically remove the fragment, whose intracardiac presence and location had been further reconfirmed by preoperative transesophageal echocardiography and fluoroscopy (Movie 1).

**Movie 1:**
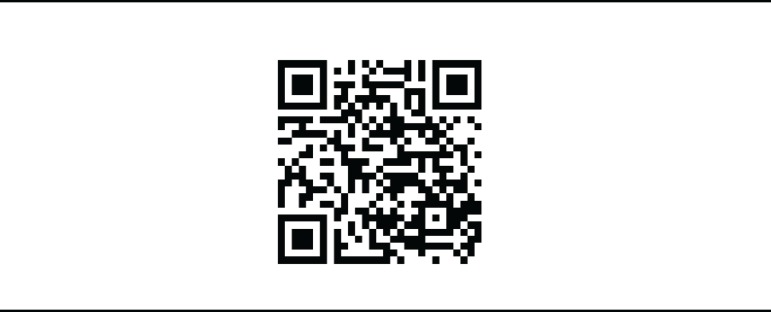
Intracardiac mobile foreign body and other fragments distributed in the thorax.

**Fig. 2:**
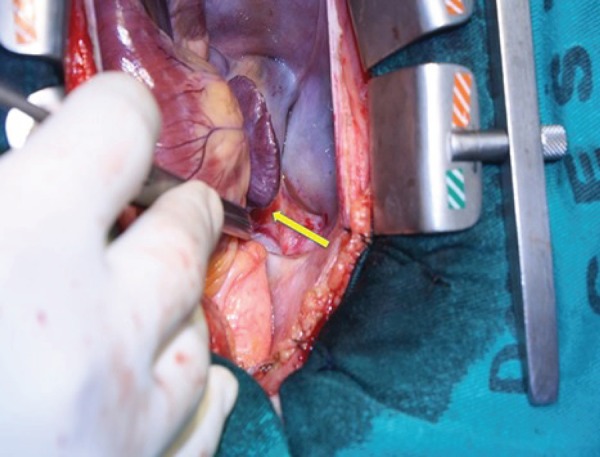
Entry location of the projectile near the right superior pulmonary vein.

The surgical approach was done by median sternotomy. During the inspection, there were no signs of pericardial effusion and the penetrating orifice was localized on the topography of the interatrial septum, between the right upper pulmonary vein and the superior vena cava (Figure 2).

After full heparinization, with 3 mg/kg intravenous unfractionated heparin, extracorporeal circulation was installed with aortic and bicaval cannulations, allowing the right atrium to be accessed. The examination of the open chamber showed no signs of presence of the projectile in this chamber or in any other location.

Fluoroscopy revealed that the fragment had migrated to the right cervical region. In order to define its precise position, arteriography of the cervical vessels was performed and showed that the projectile had lodged itself in the right external carotid artery.

**Fig. 3:**
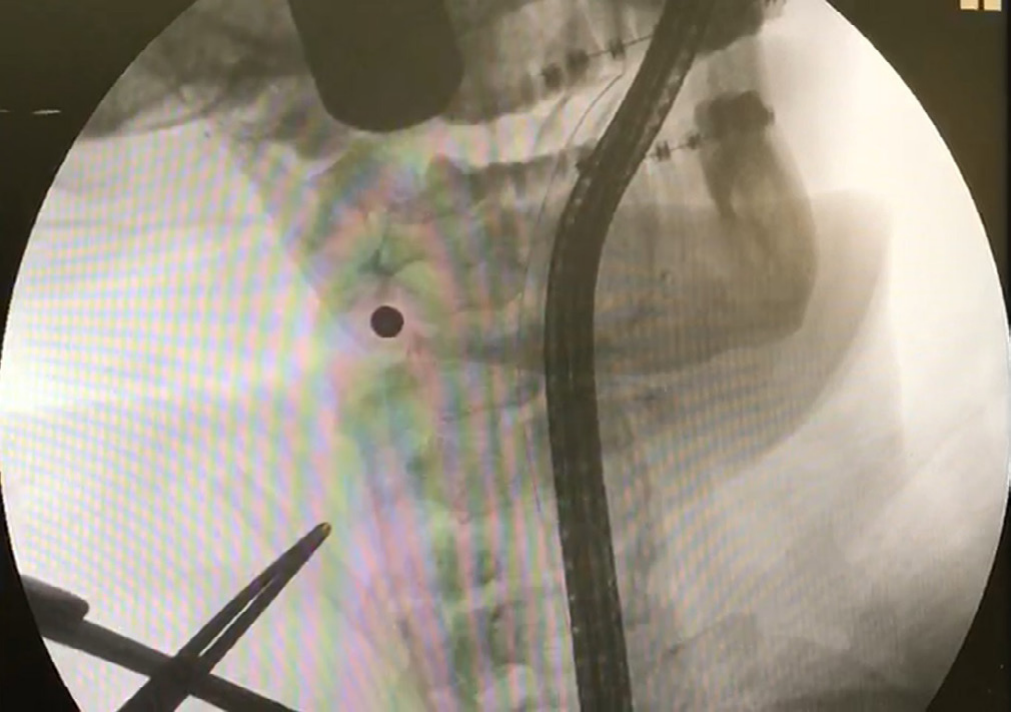
Projectile embolized and relocated in the right external carotid artery.

Angiography revealed that, although the right internal carotid artery was intact, the right external carotid artery was occluded, in its proximal portion, by the projectile (Figure 3). Because occlusion of the external carotid artery does not result in brain injury and can only cause mild facial symptoms, it was decided for a conservative treatment. Protamine was infused and the surgical procedure concluded.

The patient had no postoperative complication, and he was discharged, asymptomatic, on the fourth postoperative day. He returned to the outpatient clinic 20 days later reporting, as the only symptom, intermittent jaw claudication (episodes of involuntary movement of the mandible).

## DISCUSSION

Cases of gunshot wounds affecting the heart often have high mortality rate due to severe blood loss^[4]^. In cases of perforating injuries to the left ventricle, there is greater chance of the heart muscle sealing itself, thus preventing exsanguination^[4]^. This is less likely to occur on the right ventricle, whose thinner walls are more susceptible to perforation^[2,4]^. Cardiac tamponade and perforation of the aorta or pulmonary vein are complications also described^[3]^.

Cardiac lesions should be considered whenever there are penetrating lesions in the precordial and/or epigastric region^[2]^. TTE can provide important information^[2]^. Other imaging tests, such as radiography or CT, can be used to precisely locate the projectile^[5,6]^. The presence of metal fragments contraindicates the use of magnetic resonance in this context^[6]^.

Projectile embolization is a rare complication found in patients with penetrating injuries caused by gunshots^[3]^. The emboli usually reach the limbs, especially the left leg^[1]^. When the projectile is smaller than 3 mm, the embolization occurs, usually, in the vessels of the head and neck, where it lodges itself, preferentially, at the origin of the middle cerebral artery and it is associated with a mortality rate of 25-33%^[1]^. Embolization is symptomatic in 80% of the cases: signs of cerebral or peripheral ischaemia are valuable findings^[3]^.

Surgical intervention should be carried out when the projectile is lodged in the lumen of important vessels, especially in cases involving arteries that perfuse extremities or the extracranial internal carotid artery^[6]^. In these specific cases, the extraction of the projectile or fragment must be done at the earliest possible opportunity^[3]^.

The approach to remove the embolizing projectile may be endovascular or by open surgery, using a Fogarty catheter. Extraction by arteriotomy, after direct exposure, is recommended in case of older lesions, in which the projectile has been in situ for more than one week and it is fixed to the vessel wall^[3]^.

**Table:** 

Authors' roles & responsibilities	
ECLS	Conception and study design; realization of the operation; manuscript redaction or critical review of its content; final manuscript approval
RMT	Conception and study design; realization of the operation; manuscript redaction or critical review of its content; final manuscript approval
DLMF	Conception and study design; realization of the operation; manuscript redaction or critical review of its content; final manuscript approval
JPSPO	Conception and study design; realization of the operation; manuscript redaction or critical review of its content; final manuscript approval
FAMSF	Conception and study design; realization of the operation; manuscript redaction or critical review of its content; final manuscript approval
GAFCAL	Conception and study design; realization of the operation; manuscript redaction or critical review of its content; final manuscript approval

## References

[1] Ayad EH, Al-Wahbi AM (2005). Nail gun injury to the heart with peripheral embolization, case
report and review of the literature. Eur J Vasc Endovasc Surg.

[2] Topal AE, Celik Y, Eren MN (2010). Predictors of outcome in penetrating cardiac
injuries. J Trauma.

[3] Nguyen R, Ouedraogo A, Deneuville M (2006). Gunshot wounds to the chest with arterial bullet
embolization. Ann Vasc Surg.

[4] Asensio JA, Stewart BM, Murray J, Fox AH, Falabella A, Gomez H (1996). Penetrating cardiac injuries. Surg Clin North Am.

[5] Jodati A, Safaei N, Toufan M, Kazemi B (2011). A unique nail gun injury to the heart with a delayed
presentation. Interact Cardiovasc Thorac Surg.

[6] Dienstknecht T, Horst K, Sellei RM, Berner A, Nerlich M, Hardcastle TC (2012). Indications for bullet removal: overview of the literature, and
clinical practice guidelines for European trauma surgeons. Eur J Trauma Emerg Surg.

